# Baseline Chronic Obstructive Pulmonary Disease Identifies a High-Risk Cardiopulmonary Phenotype in Patients with Heart Failure Undergoing SGLT2 Inhibitor Therapy

**DOI:** 10.3390/diagnostics16111606

**Published:** 2026-05-25

**Authors:** Ivana Jurin, Marin Pavlov, Marin Viđak, Filip Doder, Antonio Patrk, Iva Vidaković, Nevenka Piskač Živković

**Affiliations:** 1 Clinic of Cardiology, University Hospital Dubrava, 10000 Zagreb, Croatia; marin.pavlov@gmail.com (M.P.); vidakoviciva5@gmail.com (I.V.); 2 University Center Varaždin, University North, 42000 Varaždin, Croatia; 3 Department of Internal Medicine, Division of Pulmonology, University Hospital Dubrava, 10000 Zagreb, Croatia; doderf97@gmail.com (F.D.);; 4 School of Medicine, University of Zagreb, 10000 Zagreb, Croatia; 5 Faculty of Pharmacy and Biochemistry, University of Zagreb, 10000 Zagreb, Croatia

**Keywords:** chronic obstructive pulmonary disease, heart failure, SGLT2 inhibitors, cardiopulmonary phenotype, systemic inflammation

## Abstract

**Background/Objectives:** Chronic obstructive pulmonary disease (COPD) often coexists with heart failure (HF) and can complicate the interpretation of symptoms, biomarker profiles, and clinical deterioration. Its prognostic significance at the time of sodium-glucose cotransporter 2 inhibitor (SGLT2i) initiation remains incompletely defined. We therefore evaluated whether baseline COPD was associated with a greater biomarker burden and worse 12-month outcomes in a real-world HF cohort at the time of SGLT2i initiation. **Methods:** This prospective single-centre observational cohort included patients with HF enrolled in a tertiary registry between May 2022 and November 2024 in whom SGLT2i therapy was initiated. HF was diagnosed according to contemporary European Society of Cardiology (ESC) criteria on the basis of compatible symptoms and/or signs, objective structural or functional cardiac abnormalities on echocardiography, and elevated N-terminal pro-B-type natriuretic peptide (NT-proBNP). COPD status was defined by a documented pre-existing diagnosis at baseline. The primary endpoint was the 12-month time-to-first composite of all-cause death or unplanned hospitalization for acute decompensated HF. **Results:** Among 996 patients, 122 (12.2%) had COPD. Compared with patients without COPD, those with COPD more often had a smoking history, a higher comorbidity burden, a worse New York Heart Association (NYHA) class, higher baseline N-terminal pro-B-type natriuretic peptide (NT-proBNP) and C-reactive protein (CRP) levels, and a lower estimated glomerular filtration rate (eGFR), whereas baseline HF pharmacotherapy was broadly similar. NT-proBNP remained higher at 6 and 12 months, whereas CRP remained higher at 6 months but not at 12 months. In multivariable Cox analysis adjusting for age, sex, major comorbidities, left ventricular ejection fraction (LVEF), renal function, high-density lipoprotein cholesterol (HDL-C), glycated haemoglobin (HbA1c), CRP, and log NT-proBNP, COPD remained independently associated with the primary endpoint (hazard ratio [HR] 2.610, 95% confidence interval [CI] 1.707–3.991; *p* < 0.001) and all-cause death (HR 2.097, 95% CI 1.246–3.532; *p* = 0.005). **Conclusions:** Among patients with HF starting SGLT2i therapy, baseline COPD identified a higher-risk cardiopulmonary phenotype characterized by a greater comorbidity burden, higher inflammatory and natriuretic biomarker levels, and worse 1-year outcomes. These observational findings support closer integrated cardiology–pulmonology follow-up.

## 1. Introduction

Chronic obstructive pulmonary disease (COPD) and heart failure (HF) frequently coexist in older, multimorbid patients, and this overlap has important clinical consequences. Dyspnoea, exercise intolerance, and fatigue are nonspecific symptoms that may arise from either organ system, making diagnostic assessment and longitudinal follow-up more difficult. When both diseases are present, patients generally experience more hospitalizations, more complex treatment decisions, and a worse prognosis than those with either condition alone [1,2,3].

The overlap between COPD and HF is not merely epidemiological. Both conditions are linked to smoking exposure, ageing, systemic inflammation, endothelial dysfunction, oxidative stress, and vascular disease, and each may amplify the clinical burden of the other [4,5,6]. COPD may contribute to adverse cardiopulmonary interactions through hypoxia, pulmonary vascular changes, and impaired reserve, whereas HF can aggravate respiratory symptoms through congestion and reduced pulmonary compliance.

Management is therefore challenging. In routine practice, worsening symptoms may be attributed to the wrong organ system, delaying recognition of decompensation. Clinicians may also hesitate to intensify HF therapy when chronic lung disease is present, while respiratory disease severity is often incompletely characterized in cardiology-led settings [1,2,3,7].

Sodium-glucose cotransporter 2 inhibitors (SGLT2is) are now a cornerstone of guideline-directed medical therapy for HF, with evidence of benefit across reduced, mildly reduced, and preserved ejection fraction [8,9,10,11]. Beyond their established effects on HF hospitalization and cardiovascular outcomes, these agents may also modulate inflammatory pathways and influence respiratory outcomes, including COPD exacerbation risk [12,13,14]. However, it remains uncertain whether SGLT2i initiation attenuates the adverse prognostic signal associated with coexisting COPD in real-world HF populations.

We therefore assessed whether a documented baseline diagnosis of COPD was associated with greater biomarker burden and worse 12-month outcomes after SGLT2i initiation in a real-world HF cohort.

## 2. Materials and Methods

### 2.1. Study Design and Setting

This prospective observational study was conducted at University Hospital Dubrava, Zagreb, Croatia, a tertiary HF referral centre. Consecutive patients were enrolled in the institutional HF registry between May 2022 and November 2024 after providing written informed consent. The study is reported in accordance with the Strengthening the Reporting of Observational Studies in Epidemiology (STROBE) statement. The registry prospectively captured baseline demographics, comorbidities, laboratory variables, echocardiography, treatment data, and follow-up outcomes during routine clinical care.

Treatment decisions, including the choice of dapagliflozin or empagliflozin and subsequent HF titration, were left to the treating cardiologist in accordance with contemporary European Society of Cardiology (ESC) guidance [8], local reimbursement criteria, and routine clinical practice. No protocol-specific treatment algorithm determined SGLT2i selection or later medication escalation.

### 2.2. Participants and Inclusion Criteria

Patients with HF were eligible regardless of aetiology (ischemic or non-ischemic) or clinical setting (new-onset, acute-on-chronic, inpatient, or outpatient), provided that SGLT2i therapy was initiated during the index evaluation.

HF diagnosis was established according to contemporary ESC criteria [8]. Accordingly, participants were required to have compatible symptoms and/or signs of HF, echocardiographic evidence of structural and/or functional cardiac abnormality, and elevated NT-proBNP at baseline (>300 pg/mL in patients without ongoing atrial fibrillation or flutter and >600 pg/mL in patients with ongoing atrial fibrillation or flutter). Left ventricular ejection fraction (LVEF) was assessed by the modified Simpson method by two independent echocardiographers. HF phenotypes were classified according to ESC criteria as heart failure with reduced ejection fraction (HFrEF; LVEF ≤ 40%), heart failure with mildly reduced ejection fraction (HFmrEF; LVEF 41–49%), and heart failure with preserved ejection fraction (HFpEF; LVEF ≥ 50%).

Patients in whom SGLT2i therapy was not initiated were not included. Patients in whom SGLT2i therapy was later discontinued remained in the registry and were followed according to protocol. All baseline clinical, laboratory, and echocardiographic data were obtained before SGLT2i initiation.

### 2.3. Data Collection, COPD Ascertainment, and Outcome Definitions

We collected sociodemographic characteristics, body mass index, New York Heart Association (NYHA) class, smoking history, comorbidities, laboratory values, echocardiographic variables, HF medications, hospitalizations, and mortality. Data were recorded at baseline and at the 6-month and 12-month follow-up visits within the prospective registry and were supplemented by a review of the electronic hospital record. Comorbidity burden was therefore represented by individual conditions rather than by a composite score such as the Charlson Comorbidity Index.

**Figure 1:**
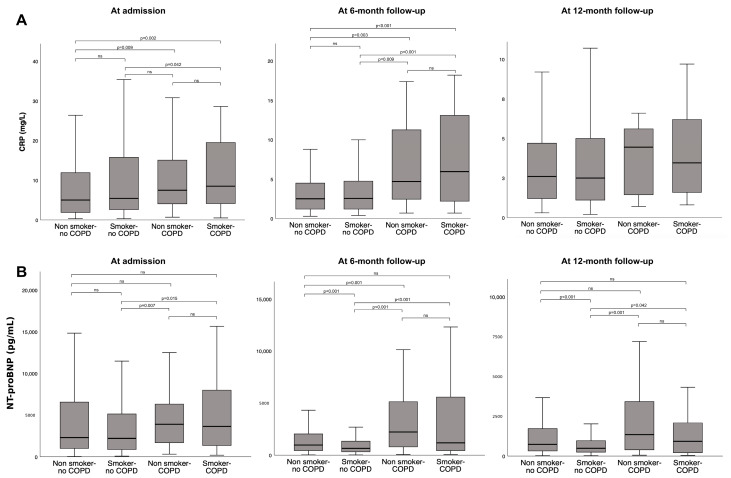
(**A**) C-reactive protein levels at study initiation and at the 6-month and 12-month follow-up according to four-way stratification by smoking history (ever-smoker [current or former smoker] versus never-smoker [no smoking history]) and COPD status; (**B**) N-terminal pro-B-type natriuretic peptide levels at the same timepoints according to the same four-way stratification. In the figure panels, the label ‘smoker’ denotes ever-smoker, and ‘non-smoker’ denotes never-smoker.

**Figure 2:**
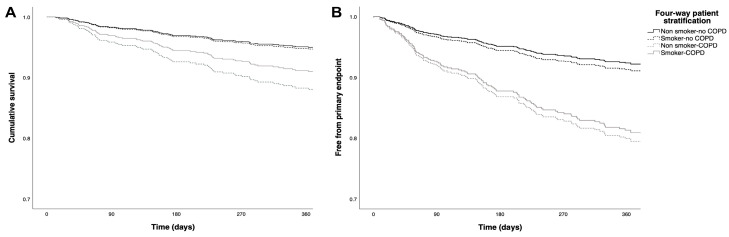
(**A**) Cumulative survival curves according to four-way stratification by smoking history (ever-smoker [current or former smoker] versus never-smoker [no smoking history]) and COPD status; (**B**) curves for freedom from the primary composite endpoint according to the same four-way stratification. In the figure panels, the label ‘smoker’ denotes ever-smoker, and ‘non-smoker’ denotes never-smoker.

Participants were grouped according to baseline COPD status. In the main comparative cohort, COPD was defined by a documented pre-existing clinical diagnosis at the index visit; post-bronchodilator spirometry was not mandated by the study protocol. In accordance with the Global Initiative for Chronic Obstructive Lung Disease (GOLD), the reference standard for COPD diagnosis is post-bronchodilator spirometry demonstrating persistent airflow obstruction (forced expiratory volume in 1 s/forced vital capacity [FEV1/FVC] < 0.70) [15]. Smoking exposure in the registry was recorded as current, former, or never smoking and was collapsed a priori into a binary smoking-history variable for subgroup analyses (ever-smoker: current or former smoker; never-smoker: no smoking history), which is the stratification used in Figure 1 and Figure 2. To further characterize respiratory status within the same 122-patient analytical COPD cohort, we performed a structured descriptive review of available specialist pulmonology records corresponding to those patients. When available, these records provided information on spirometry, GOLD stage/group, inhaled therapy, and exacerbation history; however, because this additional respiratory documentation remained incomplete and not fully standardized, it was reserved for descriptive reporting and was not used to redefine COPD status or enter the primary Cox models.

The primary outcome was the 12-month time-to-first composite of all-cause death or unplanned hospitalization for acute decompensated HF. Secondary outcomes were all-cause death and HF hospitalization, analysed separately. HF hospitalization was defined as an unscheduled hospital admission primarily attributed to acute decompensated HF, accompanied by worsening symptoms and/or signs considered by the treating team to reflect HF and requiring inpatient intensification of HF-directed therapy. When admissions in patients with COPD raised overlapping cardiorespiratory diagnostic questions, the primary discharge diagnosis and inpatient treatment course recorded in the electronic hospital record were used for event classification. Outcomes were ascertained through telephone interviews, regular outpatient follow-up, and review of electronic hospital records. For time-to-event analyses, follow-up began at SGLT2i initiation and was administratively censored at 12 months. Serial biomarker analyses used all available repeated measurements at 6 and/or 12 months.

### 2.4. Statistical Analysis

Continuous variables are presented as medians and interquartile ranges and categorical variables as counts and proportions. Distribution normality was screened with the Kolmogorov–Smirnov test, and markedly skewed variables such as NT-proBNP were log-transformed for regression analyses. Between-group comparisons at baseline or at single follow-up timepoints used the Mann–Whitney U test for continuous variables and the chi-square test for categorical variables. Associations with outcomes were evaluated using univariable and multivariable Cox proportional hazards regression analyses. For the primary composite endpoint, time-to-event analyses used the first qualifying event; accordingly, a patient hospitalized for HF and dying later contributed the date of the first HF hospitalization to the composite endpoint analysis. In the separate all-cause mortality analysis, patients were censored at 12 months if they were alive. In the separate HF-hospitalization analysis, patients were censored at death or at 12 months if no qualifying HF admission had occurred.

Covariates for multivariable modelling were prespecified on the basis of clinical relevance and data completeness and included sex, age, history of stroke, atrial fibrillation or flutter, COPD, peripheral artery disease, prior hospitalization for acute coronary syndrome, NYHA class III/IV, body mass index, high-density lipoprotein cholesterol, LVEF, estimated glomerular filtration rate, CRP, glycated haemoglobin, and log NT-proBNP. Chloride was examined only in a sensitivity model because of greater missingness. The primary Cox models were fitted in the full comparative cohort, whereas subset-level respiratory variables were reserved for descriptive and exploratory analyses. Because serial biomarker availability declined during follow-up, the primary longitudinal presentation retained available-case between-group comparisons at each time point. Additional within-patient longitudinal analyses were undertaken in patients with repeated biomarker measurements available from pulmonology follow-up. Baseline-to-6-month and baseline-to-12-month changes in NT-proBNP and CRP were assessed with Wilcoxon signed-rank tests using all available pairs. In patients with complete baseline, 6-month, and 12-month biomarker data, the Friedman test was used to explore an overall time effect across the three visits. These longitudinal analyses were exploratory and complemented, rather than replaced, the prespecified cohort-level comparisons. Missing serial values were not imputed; each analysis used the maximum number of complete cases available for the relevant variable and time point. Multivariable models were built with a forward conditional approach. A two-sided *p*-value < 0.05 was considered statistically significant. Primary analyses were performed in IBM SPSS Statistics version 30.0 (IBM Corp., Armonk, NY, USA).

### 2.5. Ethics

This study was approved by the Ethics Committee of University Hospital Dubrava (Klinička bolnica Dubrava), Zagreb, Croatia (approval code: 2022/1403-01; date of approval: 14 March 2022). Written informed consent was obtained from all participants in accordance with the approved protocol.

## 3. Results

A total of 996 patients were included in the analysis. The population was predominantly male (67.3%), with a median age of 70 (62–76) years. In total, 122 patients (12.2%) had an established diagnosis of COPD. HFrEF was the prevailing phenotype in both groups (56.6% in COPD vs. 60.9% without COPD), and the median LVEF was similar.

Compared with patients without COPD, those with COPD had a higher burden of comorbidities, including hypertension, diabetes, and peripheral artery disease. They more often presented with NYHA class III or IV symptoms and were more likely to have a smoking history. Baseline NT-proBNP and CRP levels were higher, and eGFR was lower in the COPD group. Core baseline HF pharmacotherapy was otherwise broadly similar between groups, including beta-blockers, angiotensin-converting enzyme inhibitors or angiotensin receptor blockers (ACEis/ARBs), angiotensin receptor neprilysin inhibitors (ARNIs), and mineralocorticoid receptor antagonists (MRAs), although dapagliflozin was used more often in patients with COPD (Table 1).

To further characterize respiratory status within the same analytical COPD cohort, we performed a descriptive review of available specialist pulmonology records corresponding to the 122 registry-defined COPD patients. Spirometry was available in 99/122 (81.1%), GOLD stage/group classification in 57/122 (46.7%), and documented inhaler data in 119/122 (97.5%). Among patients with documented inhaler data, 113/119 (95.0%) were receiving at least one inhaled therapy at baseline: LABA 78/119 (65.5%), ICS 58/119 (48.7%), LAMA 57/119 (47.9%), SAMA 23/119 (19.3%), SABA 14/119 (11.8%), nebulized SABA/SAMA 8/119 (6.7%), and LABA/LAMA/ICS triple therapy 26/119 (21.8%). At least one recorded exacerbation was documented in 53/122 (43.4%). These respiratory variables were descriptive only and were not used in the primary comparative models.

In patients with COPD, NT-proBNP remained higher at both the 6-month and 12-month follow-up assessments, whereas CRP remained higher at 6 months but not at 12 months (Table 2). Four-way stratification according to smoking history, defined as ever-smoker (current or former smoker) versus never-smoker (no smoking history), is presented in Figure 1.

In exploratory within-patient longitudinal analyses, NT-proBNP declined significantly from baseline to 6 months (paired *n* = 98; median 3882 vs. 1459 pg/mL; Wilcoxon *p* < 0.001) and from baseline to 12 months (paired *n* = 72; median 3561 vs. 1056.5 pg/mL; *p* < 0.001). Among the 70 patients with complete measurements at all three time points, the Friedman test likewise supported an overall time effect for NT-proBNP (*p* < 0.001). By contrast, paired CRP values trended downward but did not reach significance in the non-parametric paired analyses (baseline vs. 6 months: *n* = 49, *p* = 0.096; baseline vs. 12 months: *n* = 31, *p* = 0.112), and the Friedman test was likewise non-significant in the 21 patients with all three measurements (*p* = 0.156). These exploratory analyses complemented, rather than replaced, the prespecified cohort-level between-group comparisons reported in Table 2.

Formal longitudinal comparisons of HF-treatment intensification or inhaled therapy escalation were not feasible because follow-up treatment changes were not captured in a sufficiently standardized manner across the analytical COPD cohort.

The primary composite endpoint and each of its individual components occurred more frequently in patients with COPD (Table 1).

Several Cox regression models were fitted using a forward conditional approach. In the multivariable model including 15 variables (sex, age, history of stroke, atrial fibrillation or flutter [AFib], COPD, peripheral artery disease [PAD], prior hospitalization for acute coronary syndrome [ACS], NYHA class III/IV, body mass index, high-density lipoprotein cholesterol, LVEF, estimated glomerular filtration rate, CRP, glycated haemoglobin [HbA1c], and log NT-proBNP), all-cause death was independently associated with age (HR 1.063; 95% CI 1.035–1.093; *p* < 0.001), high-density lipoprotein cholesterol (HR 0.241; 95% CI 0.106–0.546; *p* < 0.001), history of stroke (HR 2.098; 95% CI 1.177–3.739; *p* = 0.012), log NT-proBNP (HR 1.591; 95% CI 1.281–1.975; *p* < 0.001), and COPD (HR 2.097; 95% CI 1.246–3.532; *p* = 0.005).

In the corresponding model for the primary composite endpoint, age (HR 1.042; 95% CI 1.020–1.064; *p* < 0.001), HDL cholesterol (HR 0.270; 95% CI 0.139–0.527; *p* < 0.001), history of stroke (HR 2.531; 95% CI 1.580–4.054; *p* < 0.001), log NT-proBNP (HR 1.676; 95% CI 1.402–2.004; *p* < 0.001), and COPD (HR 2.610; 95% CI 1.707–3.991; *p* < 0.001) remained independent predictors. Importantly, the association between COPD and outcome persisted despite adjustment for LVEF and major comorbidities. Similar results were observed with four-way stratification of the study population. Survival curves are shown in Figure 2.

Chloride was excluded from the primary models because of a higher proportion of missing data (30.8%). When chloride was forced into the model as a sixteenth covariate, reducing the analyzable cohort to 61.2% of all cases, COPD was no longer an independent predictor of all-cause death but remained a predictor of the primary outcome. This exploratory finding should be interpreted cautiously, given the degree of missingness.

## 4. Discussion

In this prospective observational cohort of 996 patients with heart failure (HF) at the time of sodium-glucose cotransporter 2 inhibitor (SGLT2i) initiation, coexisting chronic obstructive pulmonary disease (COPD) identified a clinically distinct high-risk subgroup. Patients with COPD had a heavier comorbidity burden, more advanced symptoms, higher CRP at baseline and 6 months, persistently higher NT-proBNP throughout follow-up, and significantly worse 1-year outcomes. Importantly, COPD remained independently associated with all-cause death and with the composite endpoint after adjustment for age, LVEF, NT-proBNP, renal function, and major comorbidities.

Our findings are consistent with previous work showing that HF-COPD overlap is associated with worse prognosis, more frequent hospital use, and more complicated day-to-day management [1,2,3,7,16,17,18]. The present study extends this literature by focusing on a contemporary real-world HF cohort at a standardized treatment milestone, namely cohort entry at SGLT2i initiation. This design should be interpreted as an observational context rather than as an interventional evaluation of SGLT2i efficacy.

The role of SGLT2i in the present study is therefore contextual rather than causal. The use of SGLT2i therapy defined cohort entry and reflects contemporary guideline-directed HF care [8,9,10,11]. Although experimental and clinical studies have suggested anti-inflammatory effects and possible respiratory benefits [12,13,14], our study was not designed to determine whether SGLT2i therapy modifies respiratory status or outcomes according to COPD status. Rather, the data indicate that documented COPD continued to identify a higher-risk phenotype within a treatment pathway that included SGLT2i.

These observations highlight the importance of integrated cardiology–pulmonology care. In patients with coexisting HF and COPD, dyspnoea, cough, wheeze, fatigue, exercise intolerance, and biomarker abnormalities rarely arise from a single isolated process. Persistent symptoms after apparent HF stabilization may justify respiratory reassessment, whereas worsening respiratory complaints in COPD should not automatically be attributed to lung disease alone. A shared follow-up framework that integrates natriuretic peptides, congestion assessment, echocardiography, oxygenation, inhaler adherence, exacerbation history, and pulmonary function testing, when clinically feasible, may improve diagnostic precision.

Additional respiratory characterization within the analytical COPD cohort suggests that these patients carried a clinically meaningful pulmonary disease burden rather than representing a loosely defined comorbidity subgroup. The high baseline use of inhaled therapy and the frequent history of exacerbations point to established chronic airway disease, whereas the incomplete availability of spirometry and GOLD classification reflects the limitations of respiratory phenotyping in routine HF care. This combination is clinically relevant because it can complicate the interpretation of dyspnoea and decompensation episodes and supports a more integrated cardiology-pulmonology approach in patients with overlapping HF and COPD.

The COPD group also had a higher burden of hypertension, diabetes, peripheral artery disease, and smoking history. Smoking almost certainly contributes to part of this excess multimorbidity burden, particularly with respect to vascular disease, but it is unlikely to be the sole explanation. COPD remained independently associated with outcome after multivariable adjustment, and in the four-way stratification, the most adverse pattern clustered with established COPD rather than with smoking history alone. This supports the concept that clinically manifest COPD identifies a broader cardiopulmonary vulnerability rather than only a marker of tobacco exposure.

It is also notable that the worse outcomes in COPD were not obviously explained by less intensive baseline HF pharmacotherapy. Core baseline guideline-directed medical therapy (GDMT) components, including beta-blockers, ACEi or ARB, ARNI, MRA, and SGLT2i initiation, were broadly similar between groups, which is reassuring because therapeutic hesitation still exists in patients with concomitant lung disease. However, longitudinal comparative titration data were not captured in a sufficiently standardized format across the cohort. Accordingly, these results should not be interpreted as evidence of differential treatment intensification during follow-up.

The biomarker trajectory in our cohort offers additional descriptive insight. Persistently higher NT-proBNP at 6 and 12 months suggests sustained haemodynamic stress or incomplete risk normalization during follow-up, while higher CRP at baseline and 6 months supports a contribution of ongoing systemic inflammation [19]. The exploratory longitudinal analyses suggest that the largest NT-proBNP improvement occurred between baseline and 6 months, with a smaller subsequent change thereafter; clinically, this still identifies a subgroup in whom biomarker normalization remained incomplete over time. Lower chloride in the COPD group may reflect a more congested, more diuretic-exposed, or more metabolically fragile phenotype, although this observation should be considered exploratory because of missing data. Troponin was not systematically available in the registry and therefore could not be used to refine myocardial injury risk in the present analysis.

The distribution of the HF phenotype also deserves comment. HFrEF predominated in both groups, median LVEF was similar, and COPD remained independently associated with outcome even after LVEF adjustment. This suggests that the observed excess risk was not explained simply by a different LVEF profile. Nevertheless, the registry was not designed for a detailed phenotype-specific comparison across HFrEF, HFmrEF, and HFpEF, and those subgroup analyses should be addressed in future studies with more granular phenotyping.

Several clinically relevant mediators could not be explored in detail. Even after restricting the descriptive respiratory review to the same 122 analytical COPD patients, GOLD severity classification was available in fewer than half of the patients, and asthma overlap could not be reliably separated in all cases, precluding formal severity-adjusted analyses. We also lacked systematic data on pulmonary hypertension or right-sided haemodynamics, troponin, bronchiectasis, and prospectively adjudicated severe aortic stenosis.

Taken together, our findings suggest that documented COPD identifies a clinically recognizable high-risk cardiopulmonary phenotype among patients with HF starting SGLT2i therapy. In this population, recurrent dyspnoea, biomarker elevation, and interval clinical deterioration warrant careful joint cardiac and respiratory interpretation. These observational data support a closer integrated assessment, but they should not be interpreted as evidence of a treatment effect.

### Limitations

This study has several limitations. First, its observational single-centre design precludes causal inference and limits generalizability. Second, COPD status in the main comparative cohort was derived from documented medical history rather than protocolized post-bronchodilator spirometry. Although we restricted the additional respiratory review to the same 122 analytical COPD patients, spirometric confirmation and severity phenotyping remained incomplete (spirometry available in 99/122; GOLD classification in 57/122), and comparable pulmonary variables were not available for the non-COPD controls. Third, outcome classification relied on real-world clinical records without central blinded adjudication. Because acute exacerbations of COPD or respiratory infections may mimic or coexist with HF decompensation, some degree of event misclassification cannot be fully excluded despite the use of the primary discharge diagnosis and inpatient treatment course to classify HF hospitalizations. Fourth, serial biomarker measurements declined over time and were not complete across all visits, so the paired Wilcoxon and Friedman analyses should be interpreted as exploratory longitudinal assessments rather than definitive longitudinal modelling across the whole cohort. Fifth, longitudinal titration of HF therapy and escalation of inhaled therapy were not captured in a sufficiently standardized format for formal comparative analysis. Sixth, several potentially relevant variables could not be fully addressed: asthma overlap could not be reliably separated in all cases, while pulmonary hypertension or right-sided haemodynamics, troponin, bronchiectasis, and systematically adjudicated severe aortic stenosis were not uniformly recorded. Finally, the cohort was derived from a tertiary HF centre and was predominantly composed of patients with HFrEF, which may limit applicability to broader HF populations and to phenotype-specific inference.

## 5. Conclusions

In conclusion, baseline COPD in patients with HF undergoing SGLT2i therapy identified a high-risk cardiopulmonary phenotype characterized by a greater comorbidity burden, higher inflammatory and natriuretic biomarker levels, and worse 1-year outcomes. The association persisted after adjustment for LVEF and major comorbidities. These findings suggest that documented COPD warrants careful cardiopulmonary phenotyping and integrated follow-up, while prospective studies with protocolized spirometry and adjudicated outcomes are needed.

## Figures and Tables

**Table 1:** Baseline characteristics, laboratory variables, treatment, and outcomes according to COPD status.

Variable	COPD (*n* = 122)	No COPD (*n* = 874)	*p*-Value
**Demographics and Clinical Presentation**			
Female sex	32 (26.2%)	294 (33.6%)	0.102
Age, years	71 (65–77)	69 (61–76)	0.052
Heart failure with reduced ejection fraction	69 (56.6%)	532 (60.9%)	0.362
NYHA class III-IV	71 (58.2%)	379 (43.4%)	0.002
LVEF, %	40 (30–50)	40 (30–48)	0.677
Acute coronary syndrome	15 (12.3%)	146 (16.7%)	0.213
Index admission for HF	94 (77.0%)	600 (68.6%)	0.059
ICU admission during index hospitalization	20 (16.4%)	205 (23.5%)	0.081
Systolic blood pressure, mmHg	130 (112–141)	130 (120–150)	0.081
Diastolic blood pressure, mmHg	80 (70–89)	80 (75–90)	0.006
Heart rate at admission, beats/min	81 (71–96)	82 (70–100)	0.510
Heart rate at discharge, beats/min	77 (68–88)	77 (67–87)	0.373
BMI, kg/m^2^	29.8 (25.8–34.6)	28.7 (25.7–32.5)	0.060
**Medical history and comorbidities**			
Hypertension	116 (95.1%)	748 (85.6%)	0.004
Smoking history	66 (54.1%)	279 (31.9%)	<0.001
Dyslipidaemia	88 (72.7%)	626 (71.6%)	0.801
Diabetes	90 (73.8%)	561 (64.4%)	0.042
Coronary artery disease	64 (57.1%)	458 (53.6%)	0.475
Stroke	6 (4.9%)	91 (10.4%)	0.055
Peripheral artery disease	36 (29.5%)	147 (16.8%)	0.001
Atrial fibrillation/flutter	65 (53.3%)	481 (55.0%)	0.715
**Baseline laboratory variables**			
NT-proBNP, pg/mL	3722 (1558–7420)	2279 (988–6059)	0.004
Hemoglobin on admission, g/L	135 (120–148)	137 (126–148)	0.145
eGFR, mL/min/1.73 m^2^	62.2 (42.6–78.6)	66.4 (48.9–84.4)	0.024
Glucose on admission, mmol/L	7.1 (5.6–9.3)	6.6 (5.7–8.7)	0.551
CRP, mg/L	7.8 (4.1–18.2)	5.3 (2.1–13.5)	<0.001
Albumin, g/L	39 (36–42)	40 (37–43)	0.076
HbA1c, %	6.4 (5.9–7.2)	6.2 (5.8–6.8)	0.006
Chloride, mmol/L	100 (98–104)	103 (100–105)	<0.001
Total cholesterol, mmol/L	4.4 (3.3–5.1)	4.3 (3.5–5.3)	0.693
LDL cholesterol, mmol/L	2.6 (1.8–3.2)	2.5 (1.8–3.4)	0.630
HDL cholesterol, mmol/L	1.1 (0.9–1.3)	1.2 (1–1.4)	0.331
Triglycerides, mmol/L	1.2 (1–1.7)	1.2 (0.9–1.6)	0.548
**Heart failure treatment**			
Dapagliflozin/empagliflozin	76/46 (62.3%/37.7%)	452/422 (51.7%/48.3%)	0.028
SGLT2i discontinued during follow-up	23 (19.5%)	130 (15.2%)	0.224
Beta-blocker therapy	107 (87.7%)	798 (91.3%)	0.196
ACEi or ARB	81 (66.4%)	540 (61.8%)	0.325
ARNI	31 (25.4%)	253 (28.9%)	0.325
MRA	96 (78.7%)	713 (81.6%)	0.444
**Follow-up data**			
NT-proBNP at 6 months, pg/mL	1354 (550–5350)	820.5 (379–1789)	<0.001
NT-proBNP at 12 months, pg/mL	1112 (363–2506)	651 (285–1439)	0.022
CRP at 6 months, mg/L	5.5 (2.2–11.6)	2.5 (1.2–4.6)	<0.001
CRP at 12 months, mg/L	3.9 (1.6–5.9)	2.6 (1.1–4.7)	0.137
LVEF at 6 months, %	41 (30–51)	45 (35–50)	0.139
LVEF at 12 months, %	45 (35–55)	45 (37–54)	0.495
Heart rate at 6 months, beats/min	75 (62–88)	70 (62–80)	0.069
Follow-up duration, days	365 (202–367)	365 (240.5–368)	0.184
**Clinical outcomes**			
All-cause death at 12 months	23 (18.9%)	83 (9.5%)	0.002
HF hospitalization at 12 months	20 (16.4%)	47 (5.4%)	<0.001
Composite endpoint at 12 months	36 (29.5%)	112 (12.8%)	<0.001

Data are presented as *n* (%) or median (interquartile range). ACEi, angiotensin-converting enzyme inhibitor; ARB, angiotensin receptor blocker; ARNI, angiotensin receptor neprilysin inhibitor; BMI, body mass index; COPD, chronic obstructive pulmonary disease; CRP, C-reactive protein; eGFR, estimated glomerular filtration rate; HbA1c, glycated haemoglobin; HDL, high-density lipoprotein; HF, heart failure; ICU, intensive care unit; LDL, low-density lipoprotein; LVEF, left ventricular ejection fraction; MRA, mineralocorticoid receptor antagonist; NT-proBNP, N-terminal pro-B-type natriuretic peptide; NYHA, New York Heart Association; SGLT2i, sodium-glucose cotransporter 2 inhibitor.

**Table 2:** CRP and NT-proBNP levels at baseline and follow-up according to COPD status.

Biomarker and Timepoint	COPD	No COPD	*p*-Value
**C-reactive protein (CRP)**			
CRP at admission, mg/L	7.8 (4.1–18.2) [105]	5.3 (2.1–13.5) [740]	<0.001
CRP at 6 months, mg/L	5.5 (2.2–11.6) [53]	2.5 (1.2–4.6) [463]	<0.001
CRP at 12 months, mg/L	3.9 (1.6–5.9) [28]	2.6 (1.1–4.7) [233]	0.137
**N-terminal pro-B-type natriuretic peptide (NT-proBNP)**			
NT-proBNP at admission, pg/mL	3722 (1558–7420) [121]	2279 (988–6059) [857]	0.004
NT-proBNP at 6 months, pg/mL	1354 (550–5350) [103]	820.5 (379–1789) [778]	<0.001
NT-proBNP at 12 months, pg/mL	1112 (363–2506) [69]	651 (285–1439) [600]	0.022

Values are presented as median (interquartile range) [available *n*]. COPD, chronic obstructive pulmonary disease; CRP, C-reactive protein; NT-proBNP, N-terminal pro-B-type natriuretic peptide.

## Data Availability

The data are not publicly available because they contain potentially identifiable clinical information. De-identified data may be made available by the corresponding author upon reasonable request and subject to institutional and ethical restrictions.

## References

[1] Le Jemtel T.H., Padeletti M., Jelic S. (2007). Diagnostic and Therapeutic Challenges in Patients with Coexistent Chronic Obstructive Pulmonary Disease and Chronic Heart Failure. J. Am. Coll. Cardiol..

[2] Canepa M., Straburzynska-Migaj E., Drozdz J., Fernandez-Vivancos C., Pinilla J.M.G., Nyolczas N., Temporelli P.L., Mebazaa A., Lainscak M., Laroche C. (2018). Characteristics, Treatments and 1-Year Prognosis of Hospitalized and Ambulatory Heart Failure Patients with Chronic Obstructive Pulmonary Disease in the European Society of Cardiology Heart Failure Long-Term Registry. Eur. J. Heart Fail..

[3] Khan S.S., Kalhan R. (2022). Comorbid Chronic Obstructive Pulmonary Disease and Heart Failure: Shared Risk Factors and Opportunities to Improve Outcomes. Ann. Am. Thorac. Soc..

[4] dos Santos N.C., Miravitlles M., Camelier A.A., de Almeida V.D.C., Maciel R.R.B.T., Camelier F.W.R. (2022). Prevalence and Impact of Comorbidities in Individuals with Chronic Obstructive Pulmonary Disease: A Systematic Review. Tuberc. Respir. Dis..

[5] King P.T. (2015). Inflammation in Chronic Obstructive Pulmonary Disease and Its Role in Cardiovascular Disease and Lung Cancer. Clin. Transl. Med..

[6] Murphy S.P., Kakkar R., McCarthy C.P., Januzzi J.L. (2020). Inflammation in Heart Failure: JACC State-of-the-Art Review. J. Am. Coll. Cardiol..

[7] Ehteshami-Afshar S., Mooney L., Dewan P., Desai A.S., Lang N.N., Lefkowitz M.P., Petrie M.C., Rizkala A.R., Rouleau J.L., Solomon S.D. (2021). Clinical Characteristics and Outcomes of Patients with Heart Failure with Reduced Ejection Fraction and Chronic Obstructive Pulmonary Disease: Insights from PARADIGM-HF. J. Am. Heart Assoc..

[8] McDonagh T.A., Metra M., Adamo M., Gardner R.S., Baumbach A., Böhm M., Burri H., Butler J., Čelutkienė J., Chioncel O. (2021). 2021 ESC Guidelines for the Diagnosis and Treatment of Acute and Chronic Heart Failure. Eur. Heart J..

[9] McMurray J.J.V., Solomon S.D., Inzucchi S.E., Køber L., Kosiborod M.N., Martinez F.A., Ponikowski P., Sabatine M.S., Anand I.S., Bělohlávek J. (2019). Dapagliflozin in Patients with Heart Failure and Reduced Ejection Fraction. N. Engl. J. Med..

[10] Anker S.D., Butler J., Filippatos G., Ferreira J.P., Bocchi E., Böhm M., Brunner-La Rocca H.P., Choi D.J., Chopra V., Chuquiure-Valenzuela E. (2021). Empagliflozin in Heart Failure with a Preserved Ejection Fraction. N. Engl. J. Med..

[11] Solomon S.D., McMurray J.J.V., Claggett B., de Boer R.A., DeMets D., Hernandez A.F., Inzucchi S.E., Kosiborod M.N., Lam C.S.P., Martinez F. (2022). Dapagliflozin in Heart Failure with Mildly Reduced or Preserved Ejection Fraction. N. Engl. J. Med..

[12] Theofilis P., Sagris M., Oikonomou E., Antonopoulos A.S., Siasos G., Tsioufis K., Tousoulis D. (2022). The Impact of SGLT2 Inhibitors on Inflammation: A Systematic Review and Meta-Analysis of Studies in Rodents. Int. Immunopharmacol..

[13] Gupta S., Mohta A., Lauinger A., Thameem D. (2024). The Role of Sodium-Glucose Transporter-2 Inhibitors (SGLT-2i) in Preventing Chronic Obstructive Disease Exacerbation in Patients with Diabetes and COPD: An Electronic Health Database Analysis. Heart Lung.

[14] Yen F.S., Wei J.C.C., Huang Y.H., Hsu T.J., Wang S.T., Hwu C.M., Hsu C.C. (2025). SGLT-2 Inhibitors and the Risk of Chronic Obstructive Pulmonary Disease Exacerbations and Mortality in Chronic Obstructive Pulmonary Disease Patients. Ann. Am. Thorac. Soc..

[15] Global Initiative for Chronic Obstructive Lung Disease (GOLD) (2024). Global Strategy for Prevention, Diagnosis and Management of COPD: 2024 Report.

[16] Mooney L., Hawkins N.M., Jhund P.S., Redfield M.M., Vaduganathan M., Desai A.S., Rouleau J.L., Minamisawa M., Shah A.M., Lefkowitz M.P. (2021). Impact of Chronic Obstructive Pulmonary Disease in Patients with Heart Failure with Preserved Ejection Fraction: Insights from PARAGON-HF. J. Am. Heart Assoc..

[17] Gulea C., Zakeri R., Quint J.K. (2022). Differences in Outcomes between Heart Failure Phenotypes in Patients with Coexistent Chronic Obstructive Pulmonary Disease: A Cohort Study. Ann. Am. Thorac. Soc..

[18] Nordon C., Simons S.O., Marshall J., Müllerová H., Pollack M., Bengtsson C., Hoti F., Lobier M., Salosensaari A., Santos A.C. (2025). The Sustained Increase of Cardiovascular Risk Following COPD Exacerbations: Meta-Analyses of the EXACOS-CV Studies. ERJ Open Res..

[19] Han E., Fritzer-Szekeres M., Szekeres T., Gehrig T., Gyongyosi M., Bergler-Klein J. (2022). Comparison of High-Sensitivity C-Reactive Protein vs C-reactive Protein for Cardiovascular Risk Prediction in Chronic Cardiac Disease. J. Appl. Lab. Med..

